# VCAM-1 complements CA-125 in detecting recurrent ovarian cancer

**DOI:** 10.1186/s12014-023-09414-z

**Published:** 2023-06-25

**Authors:** Jin Song, Lori J. Sokoll, Zhen Zhang, Daniel W. Chan

**Affiliations:** 1 Center for Biomarker Discovery and Translation, Department of Pathology, Johns Hopkins University School of Medicine, Baltimore, MD 21287 USA; 2 Department of Oncology, Johns Hopkins University School of Medicine, Baltimore, MD 21287 USA; 3 Department of Pathology, Johns Hopkins University School of Medicine, 419 North Caroline Street, Baltimore, MD 21231 USA

**Keywords:** Ovarian cancer, Recurrent, Serum, Biomarker, Multiplex immunoassay

## Abstract

**Background:**

Close to three-quarters of ovarian cancer cases are frequently diagnosed at an advanced stage, with more than 70% of them failing to respond to primary therapy and relapsing within 5 years. There is an urgent need to identify strategies for early detection of ovarian cancer recurrence, which may lead to earlier intervention and better outcomes.

**Methods:**

A customized magnetic bead-based 8-plex immunoassay was evaluated using a Bio-Plex 200 Suspension Array System. Target protein levels were analyzed in sera from 58 patients diagnosed with advanced ovarian cancer (including 34 primary and 24 recurrent tumors) and 46 healthy controls. The clinical performance of these biomarkers was evaluated individually and in combination for their ability to detect recurrent ovarian cancer.

**Results:**

An 8-plex immunoassay was evaluated with high analytical performance suitable for biomarker validation studies. Logistic regression modeling selected a two-marker panel of CA-125 and VCAM-1 that improved the performance of CA-125 alone in detecting recurrent ovarian cancer (AUC: 0.813 versus 0.700). At a fixed specificity of 83%, the two-marker panel significantly improved sensitivity in separating primary from recurrent tumors (70.8% versus 37.5%, *P* = 0.004), demonstrating that VCAM-1 was significantly complementary to CA-125 in detecting recurrent ovarian cancer.

**Conclusions:**

A two-marker panel of CA-125 and VCAM-1 showed strong diagnostic performance and improvement over the use of CA-125 alone in detecting recurrent ovarian cancer. The experimental results warrant further clinical validation to determine their role in the early detection of recurrent ovarian cancer.

**Supplementary Information:**

The online version contains supplementary material available at 10.1186/s12014-023-09414-z.

## Background

Ovarian cancer is the fifth leading cause of cancer death among women in the United States and has the highest mortality rate of all gynecologic cancers. According to the American Cancer Society, it’s estimated 19,710 new cases of ovarian cancer will be diagnosed and 13,270 women will die from the disease in 2023 [1]. Early detection of ovarian cancer is critical for successful cure of the disease, because patients with advanced disease (≥ 80% of cases) do not respond well to primary treatment (persistent or refractory cancer) and more than 70% of patients relapse within 5 years (recurrent cancer) [2]. In addition, many patients with persistent, refractory or recurrent ovarian cancer may benefit from additional cytoreduction and/or second-line systemic therapy (salvage therapy) if the relapsed ovarian cancer can be detected early [3]. However, studies of the impact of current routine follow-up protocols (i.e., scheduled clinical visits, physical examinations, serial measurements of CA-125 or other tumor markers, and radiologic tests) on survival have shown no improvement in life expectancy in 65% of patients with relapse detected 3 months (i.e., lead time) before clinical detection [4, 5]. Therefore, there is an urgent need to develop better early detection methods to stratify ovarian cancer patients, monitor treatment response, and track postoperative recurrence.

CA-125 is the most widely used tumor marker in ovarian cancer and is often considered the “gold standard”, and serum levels are currently used to monitor the response to chemotherapy, recurrence, and disease progression in ovarian cancer patients. However, the clinical implications of elevated CA-125 levels, in particular whether retreatment should be initiated based on biochemical CA-125 recurrence alone, remain controversial [4, 5]. The U.S. Food and Drug Administration (FDA) has approved three commercial serum-based multi-marker assays (OVA1 in 2009, Overa in 2016, and ROMA in 2011) for use in triaging patients with adnexal masses in conjunction with clinical evaluation (https://www.cms.gov/medicare-coverage-database/view/lcd.aspx?lcdId=38371&ver=10). CA-125, transthyretin (TT), apolipoprotein A1 (Apo-A1), beta-2-microglobulin (ß2M), transferrin (TRFR), human epididymis protein 4 (HE4), and follicle stimulating hormone (FSH) drive these algorithms to differentiate between benign and malignant disease. To date, many different biomarkers have been identified, including growth differentiation factor 15 (GDF-15), interleukin 6 (IL-6), Interleukin-6 receptor subunit alpha (IL-6 R alpha), vascular cell adhesion molecule 1 (VCAM-1), CD276 molecule (B7-H3), syndecan-1 (SDC1), and TEK receptor tyrosine kinase (Tie-2) for detection and monitoring of ovarian cancer [6–22]. Phospholipase A2 group VII (PLA2G7) was expressed in BRCA1 mutant ovarian cancer as a protective factor and potential negative regulator of the Wnt signalling pathway [23]. Some combinations of these biomarkers such as HE4, osteopontin (OPN), and GDF-15 along with CA-125 seem promising, but still lack of sufficient sensitivity or specificity for early detection of recurrent ovarian cancer [6, 7, 9, 10, 12, 13, 24–28]. In this study, we evaluated a panel of 8 biomarkers, including B7-H3, IL-6, PLA2G7, Tie-2, GDF-15, IL-6 R alpha, sSDC1, and VCAM-1 selected based on their reported relevancy to ovarian cancer and multiplexing feasibility as a customized magnetic bead-based 8-plex immunoassay. Using a Bio-Plex 200 Suspension Array System, target protein levels in sera from 58 patients diagnosed with advanced ovarian cancer (including 34 primary and 24 recurrent tumors) and 46 healthy controls were then analyzed by the 8-plex immunoassay. The clinical performance of these biomarkers was evaluated individually and in combination for their ability to discriminate recurrent from primary ovarian cancer as a first step in identifying biomarkers with potential to complement CA125 for the early detection of ovarian cancer recurrence.

## Methods

### Patient specimens

A total of 104 serum specimens archived at the Johns Hopkins Hospital were analyzed with institutional approval, which includes sera from 46 healthy women without a history of ovarian cancer and 58 patients with histologically diagnosed stage III/IV ovarian cancer (34 primary and 24 recurrent tumors). Detailed clinicopathologic characteristics of the study cohort, including age, stage and recurrence status, are shown in Table 1. Sera from the patients with primary tumors, who had no history of ovarian cancer recurrence, were collected before treatment and before surgery. Sera from the patients with recurrent tumors, who underwent primary debulking surgery followed by routine combined chemotherapy then recured, were collected before additional treatment and surgery. All serum samples were stored at −80 ºC until analysis.

**Table 1:** Clinicopathologic characteristics of the study cohort

Variables	Number (%)
Total	104
Healthy controls	46 (44.2)*
Age (years)	
Mean ± SD	49 ± 12
Range (Median)	34–78 (48)
Primary OvCa	34 (32.7)
Age (years)	
Mean ± SD	56 ± 14
Range (Median)	18–86 (58)
Stage (III/IV)	18/16
Recurrent OvCa	24 (23.1)
Age (years)	
Mean ± SD	57 ± 13
Range (Median)	33–89 (58)
Stage (III/IV)	16/8

OvCa, ovarian cancer. *, 2 cases without age information

### Reagents

The Human Magnetic Luminex Assay (LXSAHM-08), including B7-H3, IL-6, PLA2G7, Tie-2, GDF-15, IL-6 R alpha, SDC1, and VCAM-1, was purchased from R&D Systems (Minneapolis, MN). Serum CA-125 concentrations were measured using either a two-site immunoenzymometric assay on the TOSOH AIA-600 II analyzer (Tosoh Bioscience) according to the manufacturer’s protocol or an in-house CA-125 assay.

### Multiplex immunoassay

The Human Magnetic Luminex Assay was performed on the Bio-Plex 200 system according to the manufacturer’s protocols. Samples were diluted 1:2 in the calibrator diluent. Calibration curves were generated using 7 calibrators in a threefold dilution series in the calibrator diluent derived from a mixture of the highest standard points of multiple recombinant proteins. The highest standards for the recombinant proteins in the multiplex assay were 215.8, 0.7, 883.2, 169.7, 4.8, 25.6, 63.4, and 1977.9 ng/mL for B7-H3, IL-6, PLA2G7, Tie-2, GDF-15, IL-6 R alpha, SDC1, and VCAM-1, respectively. The experiment was performed in duplicate on 96-well Bio-Plex flat bottom plates. All samples were randomized with respect to their plate locations.

Calibration curves were generated using Bio-Plex Manager software version 6.1.1 with a 5-parametric (5-PL) nonlinear logistic regression curve fitting model. Assay sensitivity (limit of blank, LOB) was defined as the concentration of analyte corresponding to the median fluorescent intensity (MFI) of the background plus two standard deviations (SD) of the mean background MFI. Intra-assay precision was calculated as the coefficient of variance (%CV) on 4 replicates of pooled normal sera (S7023 from Sigma-Aldrich) on a single assay plate. Inter-assay precision was calculated as the %CV of 4 replicates. The assay working dynamic range was defined as the range between the lower limit of quantification (LLOQ) and the upper limit of quantification (ULOQ) in which an assay is both precise (intra-assay %CV ≤ 10% and inter-assay %CV ≤ 15%) and accurate (80–120% recovery).

### Data analysis

Paired serum CA-125 measurements from the two assays were available in 24 ovarian cancer patient samples with an R of 0.9931. For 15 cases where only in-house CA-125 measurements were available, values were converted to equivalent TOSOH CA-125 values. Biomarker data were log-transformed prior to analysis and further standardized in multivariate analysis. Differences between groups (primary tumors versus healthy controls, recurrent tumors versus healthy controls, and primary tumors versus recurrent tumors) were evaluated using the Kruskal–Wallis test followed by Dunn’s multiple comparisons test and Mann–Whitney U test, with a *p*-value (two-tailed) less than 0.05 considered significant. Receiver-operating-characteristic (ROC) curve analysis was performed and the area under the curve (AUC) plus its 95% confidence interval (CI) was calculated separately for each of the 9 biomarkers and the combinations of biomarkers. The Delong test was used to compare the AUCs. Logistic regression models were constructed with backward stepwise variable selection using biomarkers with an initial univariate AUC > 0.6. For the identified multivariate panels, the improvement in sensitivity (SN) at a fixed level of specificity (SP) was evaluated. The performance of the identified multivariate panels was further evaluated by Monte Carlo cross-validation (MCCV). Statistica 13 (StatSoft), GraphPad Prism 6 (GraphPad Software), MedCalc (MedCalc Software, Ostend, Belgium), and in-house developed scripts were used for statistical analysis.

## Results

A customized magnetic bead-based 8-plex immunoassay of B7-H3, IL-6, PLA2G7, Tie-2, GDF-15, IL-6 R alpha, SDC1, and VCAM-1 was evaluated using a Bio-Plex 200 Suspension Array System. Calibration curves of the 8-plex immunoassay were generated using the 5PL nonlinear logistic regression model (Additional file 1: Figure S1). The 8-plex immunoassay had acceptable analytical performance with recoveries of 98–102%, intra-assay precision of 1.2–3.3%, inter-assay precision of 1.8–8.1%, wide dynamic concentration ranges (> 2 logs) defined by LLOQ and ULOQ, and low LOBs for target protein quantification (Additional file 1: Table S1).

**Fig. 1:**
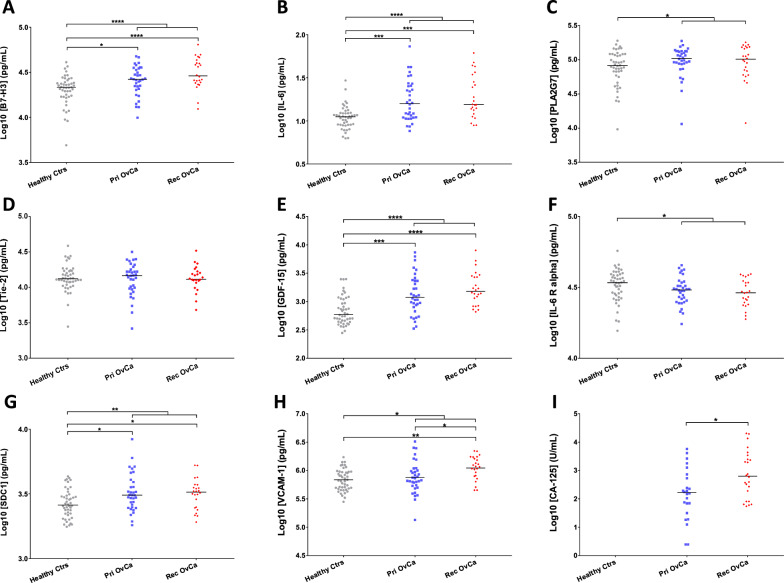
Analysis of biomarkers in sera from primary and recurrent ovarian cancer patients as well as healthy controls. **A**–**I**, Log10 transformed expression of B7-H3, IL-6, PLA2G7, Tie-1, GDF-15, IL-6 R alpha, SDC1, VCAM-1, and CA-125 in primary (Pri) and recurrent (Rec) ovarian cancer (OvCa) as well as healthy controls are demonstrated in scatterplots. Biomarkers demonstrating significant differences between Pri OvCa and Rec OvCa or between OvCa (or Pri OvCa or Rec OvCa) and healthy controls are shown with asterisks (Kruskal–Wallis test followed by Dunn’s multiple comparisons test). Bars indicate median value. *p < 0.05; **p < 0.01; ***p < 0.001; ****p < 0.0001 (two-tailed)

The 8-plex immunoassay was used to analyze the target protein levels in sera from 58 patients diagnosed with advanced ovarian cancer including 34 primary [mean (SD) age, 56 (14)] and 24 recurrent tumors [57 (13)], as well as 46 healthy controls [49 (12)] (Table 1 and Additional file 1: Table S2). As shown in Fig. 1A–H, serum levels of B7-H3, IL-6, PLA2G7, GDF-15, SDC1, and VCAM-1 were significantly increased in ovarian cancer patients compared to healthy controls (B7-H3, IL-6, and GDF-15 at *P* < 0.0001; SDC1 at *P* < 0.01; PLA2G7 and VCAM-1 at *P* < 0.05), while ovarian cancer patients had significant lower serum IL-6 R alpha levels than healthy controls (*P* < 0.05). More specifically, serum B7-H3, IL-6, GDF-15, and SDC1 levels were significantly increased in primary tumors compared to healthy controls (IL-6 and GDF-15 at *P* < 0.001; B7-H3 and SDC1 at *P* < 0.05), while serum levels of B7-H3, IL-6, GDF-15, SDC1, and VCAM-1 were also significantly increased in recurrent tumors compared to healthy controls (B7-H3 and GDF-15 at *P* < 0.0001; IL-6 at *P* < 0.001; VCAM-1 at *P* < 0.01; SDC1 at *P* < 0.05). The performance of each marker was further compared with CA-125 to discriminate between primary and recurrent tumors (Fig. 1A–I). Serum levels of VCAM-1 and CA-125 were significantly elevated in recurrent tumors compared to primary tumors (CA-125 and VCAM-1 at *P* < 0.05).

**Fig. 2:**
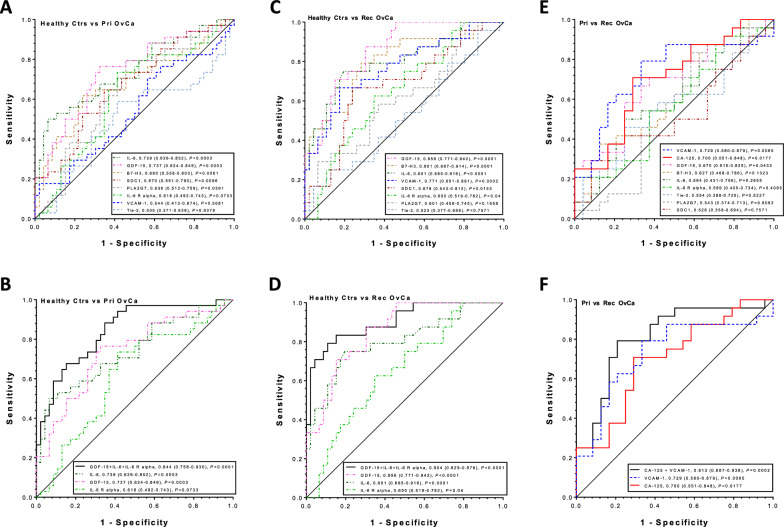
Diagnostic performance of serum biomarkers individually and combination in detecting recurrent ovarian cancer. Receiver operator characteristics (ROC) curves for B7-H3, IL-6, PLA2G7, Tie-1, GDF-15, IL-6 R alpha, SDC1, VCAM-1, and CA-125 as individual markers (**A**, **C**, **E**) and their complementary (**B**, **D**, **F**) in differentiating patients with primary ovarian cancer (Pri OvCa) versus healthy controls (**A**, **B**) or recurrent ovarian cancer (Rec OvCa) versus healthy controls (**C**, **D**) or Pri OvCa versus Rec OvCa (**E**, **F**). The area under the curve (AUC) for each marker or panel is presented along with its 95% confidence interval and *p* value in brackets

In ROC curve analysis (Fig. 2A, C and E), the 3 best individual biomarkers to separate primary tumors from healthy controls were IL-6 (AUC = 0.739, 95% CI of [0.626–0.852], *P* = 0.0003), GDF-15 (0.737, [0.624–0.849], *P* = 0.0003), and B7-H3 (0.680, [0.558–0.803], *P* = 0.0061). The 3 best biomarkers to separate recurrent tumors from healthy controls were GDF-15 (0.856, [0.771–0.942], *P* < 0.0001), B7-H3 (0.801, [0.687–0.914], *P* < 0.0001), and IL-6 (0.801, [0.685–0.916], *P* < 0.0001). The 3 best biomarkers to separate primary from recurrent tumors were VCAM-1 (0.729, [0.580–0.879], *P* = 0.0065), CA-125 (0.700, [0.551–0.848], *P* = 0.0177), and GDF-15 (0.670, [0.515–0.825], *P* = 0.0433).

In multivariate logistic regression with backward stepwise model selection (Fig. 2B, D and F; Table 2), a three-marker panel of GDF-15 (*P* = 0.037), IL-6 (*P* = 0.007), and IL-6 R alpha (*P* = 0.004) remained in the model for primary tumors versus healthy controls with an AUC of 0.844 (0.758–0.930, *P* < 0.0001), which was greater than the individual biomarkers (*P* value: GDF-15 at 0.017, IL-6 at 0.021, or IL-6 R alpha at 0.002). The same three-marker panel of GDF-15 (*P* = 0.006), IL-6 (*P* = 0.014), and IL-6 R alpha (*P* = 0.016) also remained in the model for recurrent tumors versus healthy controls with an AUC of 0.904 (0.829–0.979, *P* < 0.0001), which was greater than the individual biomarkers (*P* value: GDF-15 at 0.122, IL-6 at 0.059, or IL-6 R alpha at 0.0001). In addition, a two-marker panel of CA-125 (*P* = 0.022) and VCAM-1 (*P* = 0.028) remained in the model and had an AUC of 0.813 (0.687–0.938, *P* = 0.0002), which was greater than the individual biomarkers for primary versus recurrent tumors (AUC/*P* value: CA-125 at 0.700/0.062, VCAM-1 at 0.729/0.160), even though these were not statistically significant.

**Table 2:** Performance of individual and combined biomarkers in detecting recurrent ovarian cancer

	AUC (95% CI)	SN (%)	SP (%)
Healthy controls vs primary OvCa			
IL-6 R alpha	0.618 (0.492–0.743)	21.7^&^	85
GDF-15	0.737 (0.624–0.849)	43.5^#^	85
IL-6	0.739 (0.626–0.852)	41.3^$^	85
GDF-15 + IL-6 + IL-6 R alpha	0.844 (0.758–0.930)	65.2	85
Healthy controls vs recurrent OvCa			
IL-6 R alpha	0.650 (0.519–0.782)	30.4^&^	83
IL-6	0.801 (0.685–0.916)	50.0^&^	83
GDF-15	0.856 (0.771–0.942)	69.6^#^	83
GDF-15 + IL-6 + IL-6 R alpha	0.904 (0.829–0.979)	84.8	83
Primary vs recurrent OvCa			
CA-125	0.700 (0.551–0.848)	37.5^#^	83
VCAM-1	0.729 (0.580–0.879)	58.3	83
CA-125 + VCAM-1	0.813 (0.687–0.938)	70.8	83

*OvCA* ovarian cancer, *AUC* area under curve, *CI* confidence interval, *SN* sensitivity, *SP* specificity. One-sided McNemar test comparing sensitivity against individual markers: ^*^p < 0.05; ^#^*p* < 0.01; ^$^*p* < 0.001; ^&^*p* < 0.0001

As shown in Table 2, at a fixed SP of 85%, the three-marker panel of GDF-15, IL-6, and IL-6 R alpha significantly improved the SN in separating primary tumors from healthy controls compared to the individual biomarkers (all *P* < 0.01). At a fixed SP of 83%, the same three-marker panel of GDF-15, IL-6, and IL-6 R alpha also significantly improved the SN in separating recurrent tumors from healthy controls compared to the individual biomarkers (all *P* < 0.01). In addition, at a fixed SP of 83%, the two-marker panel of CA-125 and VCAM1 as well as VCAM-1 alone significantly improved SN in separating primary from recurrent tumors compared to CA-125 alone (70.8% versus 37.5%, *P* = 0.004 and 58.3% versus 37.5%, *P* = 0.031).

The above described comparisons were further performed through MCCV (100 repeats, 30% leave-out) and analysed by paired Wilcoxon signed rank test. The improvement of regression models over individual markers in either ROC/AUC or SN at the fixed SPs in cross-validation was significant (*P* < 0.0001) for both recurrent tumors versus healthy controls and primary versus recurrent tumors. For primary tumors versus healthy controls, the p-values for the difference in ROC/AUC and in SN was < 0.0001 and < 0.0003, respectively.

## Discussion

In this study, an 8-plex immunoassay for B7-H3, IL-6, PLA2G7, Tie-2, GDF-15, IL-6 R alpha, SDC1, and VCAM-1 was determined to have high analytical performance suitable for biomarker validation studies. It was applied to a set of serum samples from 58 patients diagnosed with advanced ovarian cancer (including 34 primary and 24 recurrent tumors) and 46 healthy controls to evaluate the performance of the eight biomarkers individually and in combination for their ability to complement CA-125 in the detection of recurrent ovarian cancer. A three-marker panel of GDF-15, IL-6, and IL-6 R alpha was initially identified to differentiate primary or recurrent ovarian cancer from healthy controls. A two-marker panel of CA-125 and VCAM-1 was further identified to detect recurrent ovarian cancer. The two-marker panel of CA-125 and VCAM-1 showed strong diagnostic performance and improvement over the use of CA-125 alone in terms of AUC (0.813 versus 0.700). At a fixed SP of 83%, the two-marker panel of CA-125 and VCAM-1 performed best in separating primary from recurrent tumors due to a significant improvement in the SN (70.8% versus 37.5%), demonstrating that VCAM-1 is complementary to CA-125 for the identification of recurrent ovarian cancer. Even though the current study could be considered as an independent evaluation of the individual biomarkers, results from the multivariate analysis were however limited due to the use of the same sample set for model selection and evaluation. The additional MCCV analysis, to some degree, helped to confirm that the observed improvements by the multivariate models over those of the component biomarkers individually remained statistically significant. Another limitation of this study was that all 24 recurrent cases included in this observational study were clinical recurrence rather than biochemical recurrence. Since survival benefit is often dependent on lead time and preclinical detection rates, further studies are needed to validate the complementarity of serum VCAM-1 and CA-125 in detecting recurrent ovarian cancer and to examine the potential role of the identified biomarker panel for the early detection of ovarian cancer recurrence.

VCAM-1 (CD106), a 90-kDa cell surface glycoprotein expressed by cytokine-activated vascular endothelial cells, was originally identified as a vascular cell adhesion molecule involved in the regulation of inflammation-associated vascular endothelial cell adhesion and signal transduction [29]. In addition to being associated with the progression of several immunological diseases such as rheumatoid arthritis and asthma [29], there has recently been increasing evidence that elevated serum or plasma soluble VCAM-1 (sVCAM-1) levels are also associated with the progression of various cancers such as breast [30, 31], ovarian [31], gastric [31–34], colorectal [35, 36], bladder cancers [31, 37, 38], prostate [39], leukemia [40], and myeloma [41], suggesting VCAM-1 as a potential therapeutic target in immunological diseases and cancer [29, 42]. Circulating levels of sVCAM-1 have been identified as a predictive biomarker for overall survival and postoperative recurrence in ovarian, prostate and colorectal cancer patients [8, 39, 43, 44]. Slack-Davis et al. reported that VCAM-1 interacts with its ligand α4β1 integrin and is involved in the regulation of mesothelial invasion and metastatic progression of ovarian cancer cells [45]. Using VCAM-1-specific imaging probes, VCAM-1 expression was identified as a potential marker of ovarian cancer peritoneal metastasis and therapeutic response to platinum-based agents [46]. In addition, VCAM-1 expression correlated with tumorigenesis and poor prognosis in ovarian cancer [47, 48], and higher preoperative serum sVCAM-1 concentrations in ovarian cancer patients were linked to early tumor recurrence or disease progression [8]. In our study, serum VCAM-1 levels were found to be significantly elevated in ovarian cancer (as well as recurrent tumors) when compared to healthy controls. Serum VCAM-1 levels were also significantly elevated in recurrent tumors when compared to primary tumors. In terms of significant improvement in the SN, the two-marker panel of CA-125 and VCAM-1 as well as VCAM-1 alone significantly outperformed CA-125 alone in separating primary from recurrent tumors.

Yurkovetsky et al. reported that, from a set of 96 candidate serum biomarkers, a four-marker panel of CA125, HE4, carcinoembryonic antigen (CEA), and VCAM-1 showed the best diagnostic performance with an SN of 86% or 93% for early-stage or late-stage ovarian cancer, respectively, at a fixed SP of 98% [13]. However, it was observd that VCAM-1 was lower in early-stage ovarian cancer compared with healthy women, and there was a significant difference between patients with early-stage and late-stage ovarian cancer in their study. It should be noted that all healthy women included in their study were postmenopausal and had an age range (mean/median) of 48–87 (57.8/56) and 48–77 (55.4/55) in the training and validation sets, respectively. Serum FSH levels were previously reported to be positively correlated with VCAM-1 levels, and both were significantly elevated in postmenopausal women when compared to premenopausal women [49]. Therefore, age and menopausal status may have contributed in part to the differences observed in the studies. Most studies conclude that elevated VCAM-1 levels are associated with tumorigenesis and metastasis [29, 42]. However, quantification of serum sVCAM-1 levels is currently unable to distinguish between sVCAM-1 derived from endothelial cells or tumor cells and VCAM-1 released from angiogenic lymphatic vessels [42].

## Conclusion

A magnetic bead-based 8-plex immunoassay was evaluated and demonstrated to have appropriate analytical performance to evaluate serum biomarkers that could complement CA-125 in the detection of recurrent ovarian cancer. The two-marker panel of CA-125 and VCAM-1 identified in this study showed strong diagnostic performance and improvement over the use of CA-125 alone. The experimental results warrant additional clinical validation to determine their role in the early detection of recurrent ovarian cancer, which may lead to earlier intervention and improved outcomes.

## Supplementary Information

**Additional file 1: Figure S1.** Calibration curves of the 8-plex immunoassay. A-H, calibration curves of B7-H3, IL-6, PLA2G7, Tie-1, GDF-15, IL-6 R alpha, SDC1, and VCAM-1 in the 8-plex immunoassay generated using the 5 parameter (5PL) logistic regression model. A.U., arbitrary units. **Table S1.** Analytical performance of the 8-plex immunoassay. **Table S2.** Statistics of individual biomarkers of the 8-plex assay in healthy controls, primary, and recurrent ovarian cancer patients.

## Data Availability

All data generated during this study are included in this published article.

## Abbreviations

B7-H3: CD276 molecule
IL-6: Interleukin 6
PLA2G7: Phospholipase A2 group VII
Tie-2: TEK receptor tyrosine kinase
GDF-15: Growth differentiation factor 15
IL-6 R alpha: Interleukin-6 receptor subunit alpha
SDC1: Syndecan-1
VCAM-1: Vascular cell adhesion molecule 1
