# Supplementary material for: VCAM-1 complements CA-125 in detecting recurrent ovarian cancer
Source: Clin Proteomics. 2023 Jun 25;20:25. doi: 10.1186/s12014-023-09414-z (PMC10291808; doi:10.1186/s12014-023-09414-z)
Supplement: Supplementary file 1 — Additional file 1: Figure S1. Calibration curves of the 8-plex immunoassay. A-H, calibration curves of B7-H3, IL-6, PLA2G7, Tie-1, GDF-15, IL-6 R alpha, SDC1, and VCAM-1 in the 8-plex immunoassay generated using the 5 parameter (5PL) logistic regression model. A.U., arbitrary units. Table S1. Analytical performance of the 8-plex immunoassay. Table S2. Statistics of individual biomarkers of the 8-plex assay in healthy controls, primary, and recurrent ovarian cancer patients. [file 12014_2023_9414_MOESM1_ESM.docx]

**Additional Figure Legends**

**Figure S1. Calibration curves of the 8-plex immunoassay.** A-H, calibration curves of B7-H3, IL-6, PLA2G7, Tie-1, GDF-15, IL-6 R alpha, SDC1, and VCAM-1 in the 8-plex immunoassay generated using the 5 parameter (5PL) logistic regression model. A.U., arbitrary units.

**Figure S1.**


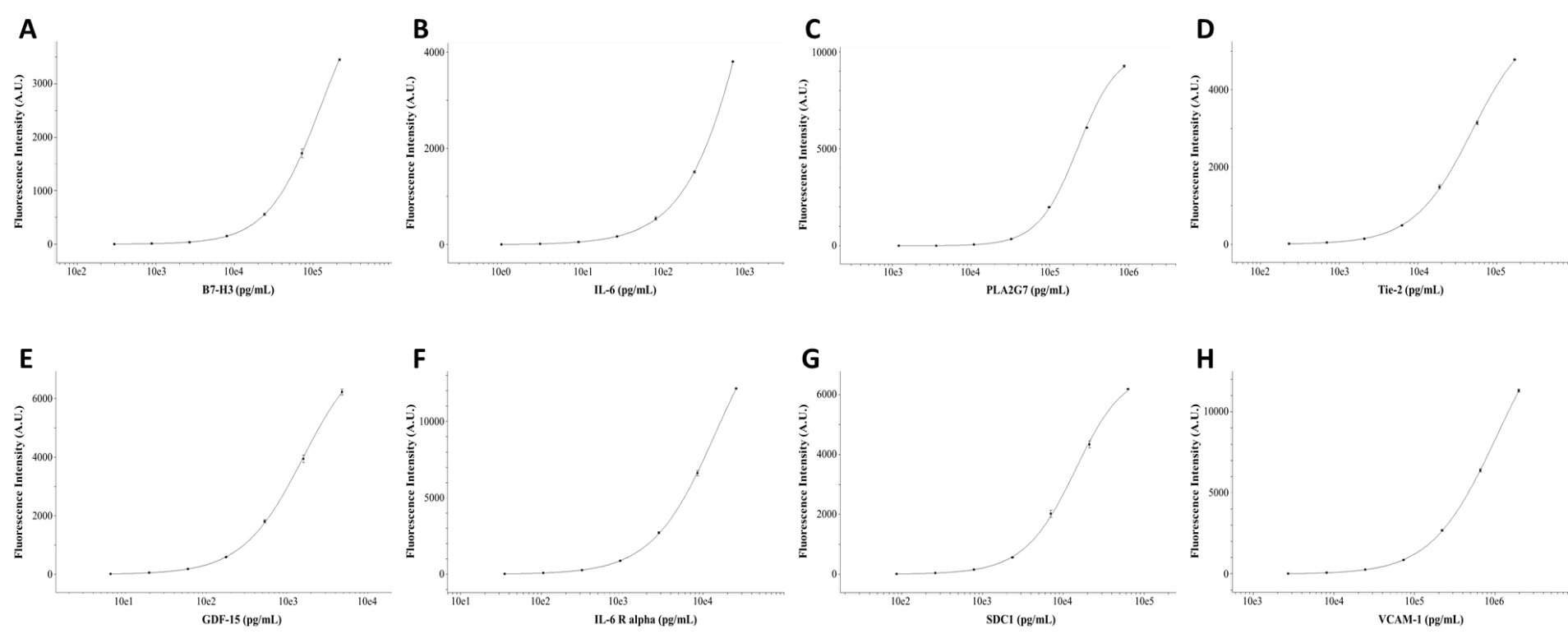


**Table S1. Analytical performance of the 8-plex immunoassay.**

|  | **Quality Control**  **Mean (pg/mL)** | | **Intra-assay Precision (%CV)** | **Inter-assay Precision (%CV)** | | | **Linearity^#^ (Pearson *R / p value*)** | **LOB (pg/mL)** | | **LLOQ (pg/mL)** | **ULOQ (pg/mL)** | | **Duplicates^*^ [%CV, mean (range)]** |
| --- | --- | --- | --- | --- | --- | --- | --- | --- | --- | --- | --- | --- | --- |
|  | **Pooled Normal**  **Serum** | | **Pooled Normal Serum** | **Pooled Normal Serum** | | |  |  | |  |  | |  |
| B7-H3 | 16315.7 | 3.1 | | | 2.9 | 0.9980/0.0020 | | | 267.4 | 283.6 | 216122.8 | 2.7 (0.0-13.2) | |
| IL-6 | 10.0 | 3.3 | | | 8.1 | 0.9996/0.0004 | | | 0.1 | 1.0 | 730.6 | 3.3 (0.0-12.6) | |
| PLA2G7 | 125530.7 | 1.7 | | | 1.8 | 0.9984/0.0016 | | | 1622.0 | 3452.1 | 899308.9 | 5.7 (0.0-21.5) | |
| Tie-2 | 9981.6 | 1.8 | | | 7.3 | 0.9994/0.0006 | | | 27.3 | 229.5 | 171957.5 | 2.2 (0.0-18.5) | |
| GDF-15 | 573.2 | 1.4 | | | 4.6 | 0.9996/0.0004 | | | 0.7 | 6.5 | 4841.6 | 2.1 (0.1-9.3) | |
| IL-6 R alpha | 27315.7 | 1.8 | | | 3.0 | 0.9999/0.0002 | | | 1.3 | 35.0 | 25711.8 | 1.5 (0.0-6.4) | |
| SDC1 | 2190.7 | 1.2 | | | 5.0 | 0.9996/0.0004 | | | 10.0 | 84.1 | 66419.8 | 2.1 (0.0-14.9) | |
| VCAM-1 | 616016.4 | 1.5 | | | 2.4 | 0.9990/0.0010 | | | 242.3 | 2717.2 | 1975251.8 | 1.6 (0.0-15.5) | |

NOTE: LOB, limit of blank. LLOQ, lower limit of quantitation. ULOQ, upper limit of quantitation. ^#^, linearity of dilution from ½ to 1/16. ^*^, mean/range of %CV for duplicates in all samples for each protein.

**Table S2. Statistics of individual biomarkers of the 8-plex assay in healthy controls, primary, and recurrent ovarian cancer patients.**

| **Biomarker** | **Subgroup** | **Number** | **Min** | **Max** | **Median** | **Mean** | **IQR** |
| --- | --- | --- | --- | --- | --- | --- | --- |
| **B7-H3** | Healthy controls | 46 | 4915.2 | 40930.8 | 21496.4 | 21500.4 | 7593.6 |
|  | Primary OvCa | 34 | 9927.9 | 47602.0 | 26400.7 | 27092.5 | 14125.1 |
|  | Recurrent OvCa | 24 | 12391.9 | 64126.4 | 28822.4 | 33135.2 | 17337.3 |
| **IL-6** | Healthy controls | 46 | 6.3 | 29.4 | 11.2 | 11.5 | 3.3 |
|  | Primary OvCa | 34 | 7.6 | 73.0 | 15.8 | 20.4 | 14.5 |
|  | Recurrent OvCa | 24 | 8.9 | 61.3 | 15.5 | 22.7 | 18.7 |
| **PLA2G7** | Healthy controls | 46 | 9601.6 | 189604.6 | 82062.1 | 87704.1 | 62141.7 |
|  | Primary OvCa | 34 | 11482.9 | 188581.3 | 104733.0 | 104976.4 | 45639.0 |
|  | Recurrent OvCa | 24 | 11834.6 | 180376.3 | 101753.9 | 104280.6 | 87317.1 |
| **Tie-2** | Healthy controls | 46 | 2789.0 | 38598.6 | 13220.1 | 14845.2 | 5221.6 |
|  | Primary OvCa | 34 | 2618.3 | 31612.2 | 14630.9 | 14643.6 | 8238.9 |
|  | Recurrent OvCa | 24 | 916.8 | 32858.8 | 13054.5 | 13923.9 | 5712.5 |
| **GDF-15** | Healthy controls | 46 | 279.2 | 2494.9 | 591.5 | 822.7 | 652.9 |
|  | Primary OvCa | 34 | 334.4 | 7375.3 | 1186.3 | 1840.4 | 1517.7 |
|  | Recurrent OvCa | 24 | 660.2 | 7998.6 | 1519.9 | 2151.4 | 1772.9 |
| **IL-6 R alpha** | Healthy controls | 46 | 15644.1 | 57268.6 | 34174.0 | 33526.1 | 11280.7 |
|  | Primary OvCa | 34 | 17454.8 | 45378.6 | 30399.3 | 30618.7 | 8721.0 |
|  | Recurrent OvCa | 24 | 18888.0 | 39267.1 | 28963.6 | 29477.5 | 10318.7 |
| **SDC1** | Healthy controls | 46 | 1759.7 | 4317.9 | 2589.7 | 2744.2 | 839.2 |
|  | Primary OvCa | 34 | 1811.9 | 8362.6 | 3089.8 | 3425.7 | 1417.6 |
|  | Recurrent OvCa | 24 | 1913.5 | 5254.6 | 3262.4 | 3256.0 | 1144.0 |
| **VCAM-1** | Healthy controls | 46 | 281487.4 | 1723500.0 | 676972.0 | 751183.0 | 464473.5 |
|  | Primary OvCa | 34 | 134296.9 | 3242700.0 | 701996.0 | 935617.0 | 465307.1 |
|  | Recurrent OvCa | 24 | 449182.2 | 2209200.0 | 1170700.0 | 1223585.0 | 867653.8 |
| **CA-125** | Healthy controls | 0 | NA | NA | NA | NA | NA |
|  | Primary OvCa | 24 | 2.5 | 5772.0 | 165.3 | 774.0 | 581.8 |
|  | Recurrent OvCa | 24 | 55.0 | 20449.0 | 631.5 | 3356.3 | 2830.5 |

NOTE: all biomarkers are at pg/mL, except CA-125 at U/mL. IQR, interquartile range. OvCA, ovarian cancer.
